# Relapse or recall? Docetaxel-associated re-emergence of paraneoplastic dermatomyositis

**DOI:** 10.1016/j.jdcr.2024.05.012

**Published:** 2024-05-18

**Authors:** Drew Kuraitis, Paul Bogner, Dharmesh Gopalakrishnan, Susan Pei

**Affiliations:** a Department of Dermatology, Roswell Park Comprehensive Cancer Center, Buffalo, New York; b Department of Dermatology, Tulane University, New Orleans, Louisiana; c Department of Medicine, Roswell Park Comprehensive Cancer Center, Buffalo, New York

**Keywords:** connective tissue disease, cutaneous recall, dermatomyositis, docetaxel, paraneoplasia, paraneoplastic dermatomyositis, paraneoplastic syndrome, recall, taxane, TIF-1γ, UV recall

## Introduction

Dermatomyositis (DM) is an inflammatory process affecting the skin and muscles. Although typically idiopathic, approximately 20% of patients may have concurrent malignancy with DM reflecting a paraneoplastic phenomenon.^1^ The presence of transcription intermediary factor-1 γ (TIF-1γ) antibodies in adults with DM is associated with underlying malignancy. Metastatic disease requires systemic therapy, many of which have associated cutaneous toxicities. Docetaxel, an antineoplastic taxane, is used for metastatic prostate cancer and is associated with numerous cutaneous toxicities, including induction of connective tissue disease (CTD), such as DM, and ultraviolet radiation (UVR) recall. In this report, we describe a patient with paraneoplastic DM in remission who developed re-emergent cutaneous DM with each docetaxel infusion, representing a drug-induced relapse or a recall effect.

## Case presentation

**Fig 1:**
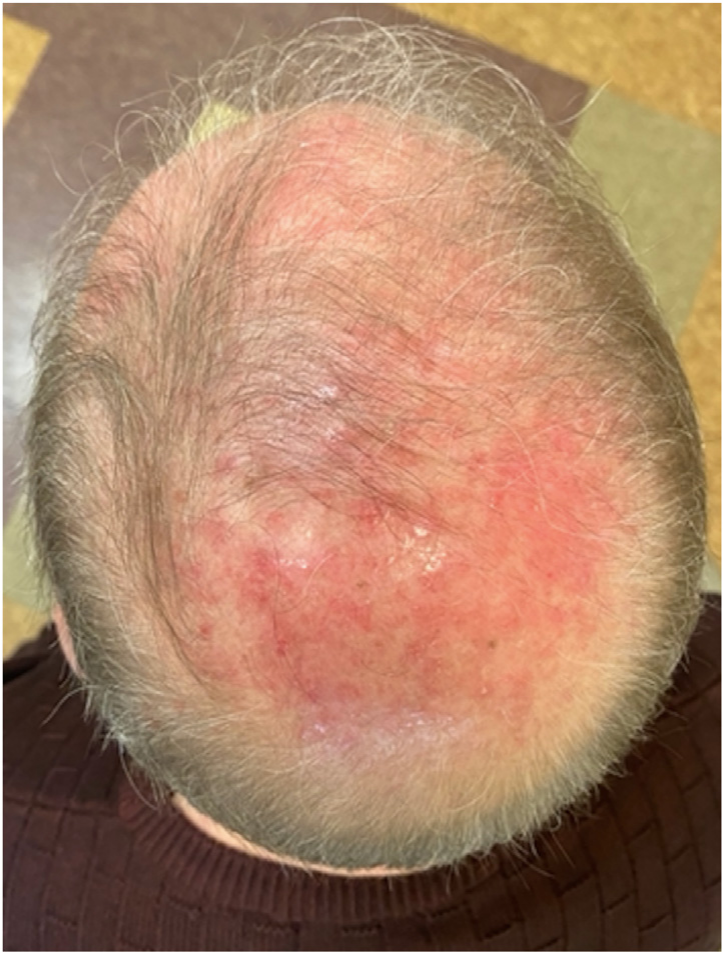
Non-scaling erythematous papules coalescing into plaques on the scalp after first infusion with docetaxel.

**Fig 2:**
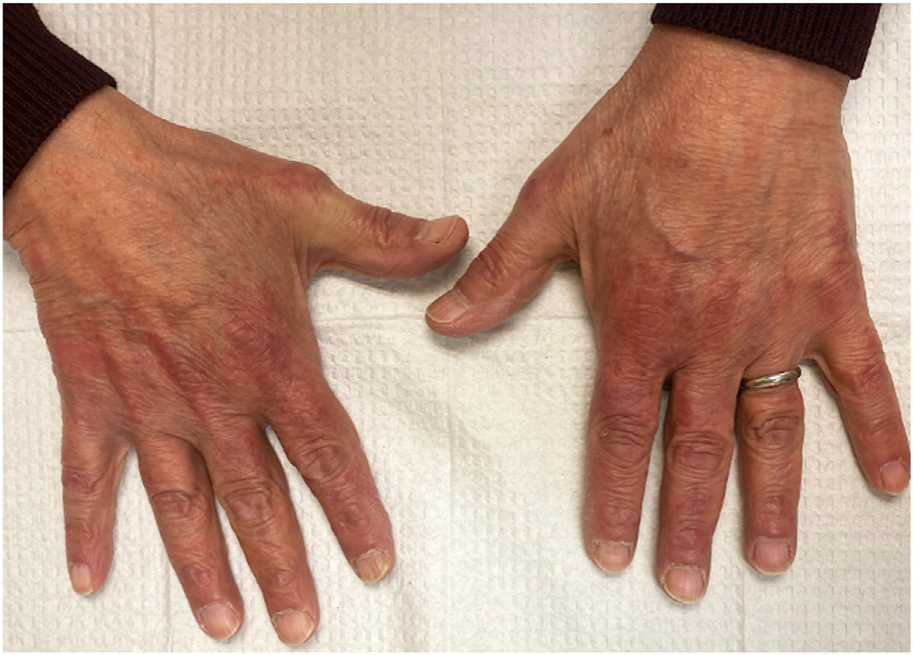
Erythema over the dorsal hands in the same location as initial dermatomyositis rash after first infusion with docetaxel.

**Fig 3:**
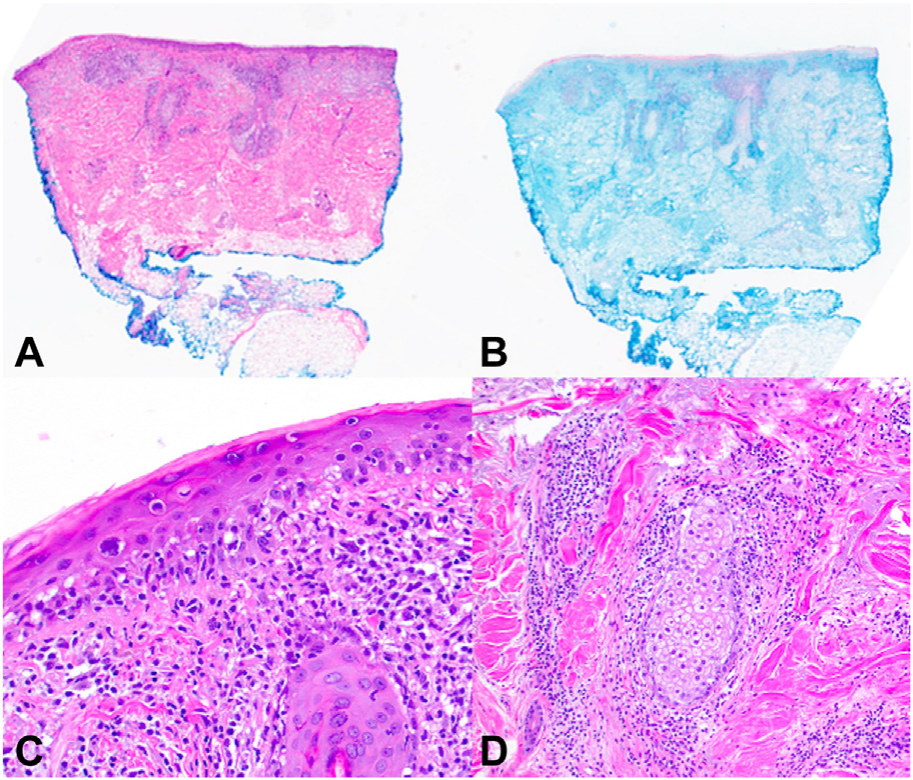
Histopathology of docetaxel-associated eruption, demonstrating vacuolar interface with a superficial and deep perivascular lymphohistiocytic infiltrate involving follicles (**A**), and increased mucin deposition in the reticular dermis, highlighted by colloidal iron (**B**). Focused evaluation of scalp biopsy demonstrating interface dermatitis (**C**; 200×) and perivascular and periadnexal inflammatory infiltrate (**D**; 100×).

**Fig 4:**
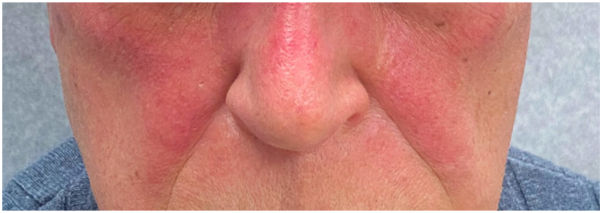
Erythema of the face after second infusion with docetaxel, involving the nasolabial folds.

A 68-year-old man presented to an outside dermatology clinic with a 2-month history of a rash with associated muscle weakness of the arms. Biopsy demonstrated interface dermatitis with a superficial and deep perivascular lymphohistiocytic infiltrate. Hemoccult was negative within the past year, and he followed with urology for prostatic hyperplasia, with prostate-specific antigen of 0.7-0.9 ng/mL in 3 preceding years (within normal limits). He was started on prednisone, hydroxychloroquine, and topical steroids for DM, and referred to our institution. Baseline photos are unavailable. Cutaneous and muscle symptoms involvement improved while on prednisone, and he started intravenous immunoglobulin (IVIG) 2 g/kg monthly. A myositis panel that evaluated for antibodies to Jo-1, PL-7, PL-12, EJ, OJ, SRP, Mi-2, TIF-1γ, MDA-5, NXP-2, sAE1, Scl-100, Ku, SSA, U1 RNP, U2 RNP, and U3 RNP yielded TIF1-1γ positivity and subsequent whole-body positron emission tomography-computed tomorgraphy imaging revealed metastatic bony lesions. Bone biopsy demonstrated poorly differentiated prostatic adenocarcinoma. Further work-up defined a Gleason 5 + 4 = 9 prostate adenocarcinoma without neuroendocrine markers on immunohistochemistry presenting with *de novo* high volume prostate-specific membrane antigen-expressing skeletal and lymph node metastases. During this time, his skin and muscle symptoms responded to the addition of IVIG and achieved remission. Three months after DM remission, he started docetaxel (75 mg/m^2^) every 21 days. After the first infusion, he developed brightly erythematous papules and plaques to his scalp (Fig 1) and hands (Fig 2), mimicking his original DM rash. Biopsy of the scalp and hand revealed interface dermatitis with superficial and deep lymphohistiocytic infiltrates (Fig 3, *A*) with increased mucin deposition (Fig 3, *B*). Due to the asymptomatic nature, he did not maintain topical steroid use and the rash faded between infusions. After the second docetaxel infusion, he also developed facial erythema that involved the nasolabial folds (Fig 4). Identical rashes recurred on the scalp, hands, and face with each of the subsequent 6 docetaxel infusions, although with variable severity each time, and faded between treatments without dermatologic intervention beyond IVIG maintenance. During each of these flares, he did not have a recurrence of symptomatic myositis or significantly elevated creatinine kinase, nor were there capillary nail fold changes. He had an excellent tumor response to chemotherapy and has been on oral antiandrogen maintenance therapy for 11 months (since completion of docetaxel until submission of this report) and has been tapering from IVIG with reduced dosing frequency, with plans to discontinue. At the time of manuscript submission, he has been without recurrence of his cutaneous DM or malignancy.

## Discussion

Of the diverse cutaneous toxicities associated with taxanes,^2^ a dermal hypersensitivity was suspected when biopsies were performed with the patient’s re-emergent rash, although unusually restricted to his previous sites of cutaneous DM. Notably, his cutaneous DM had been in remission for 3 months without flares before starting docetaxel. The histopathology of interface dermatitis with superficial and deep lymphocytic inflammation and increased dermal mucin is consistent with DM. Dermal hypersensitivity histopathology lacks interface change and demonstrates eosinophils, which would not be seen in DM. The patient’s biopsies did not show histopathologic findings of UVR (epidermal dysplasia, apoptotic keratinocytes, dermal fibrosis, vasodilation, and atypical fibroblasts).^3^ Given the re-emergent distribution and histopathologic findings identical to his initial DM eruption, in addition to facial and nasolabial fold erythema, a diagnosis of docetaxel-associated DM was rendered, although whether this is a relapse due to drug (drug-induced) or a recall effect was unclear.

Taxanes have been reported to induce CTD, particularly subacute cutaneous lupus erythematosus,^2^ and rarely DM. Taxane-induced subacute cutaneous lupus erythematosus lesions are expected to regress upon cessation of chemotherapy,^2^ although a similar body of literature regarding DM is not available. It is possible that our patient’s recurrent DM lesions were purely drug-induced, and this would fit given his cyclic presentation after each docetaxel infusion, with resolution and remission after completing chemotherapy; however, we also considered that his cyclic recurrence may represent a memory phenomenon given the fixed recurrence with each infusion mimicking his initial presentation, without recurrent myositis.

Tissue-resident memory T-cells (TRMs) develop after a T-cell-mediated immune response and persist in local tissues, mediating autoimmune, tumor, and infectious responses. TRMs are implicated in fixed drug eruptions, whereby recurrent lesions occur in identical distributions after exposure to causative medications, and TRMs have also been implicated in the pathogenesis of CTDs.^4^ Radiation recall dermatitis is the phenomenon by which a previously irradiated field develops a recurrent radiation-like dermatitis after administration of chemotherapy, including taxanes.^5^ The exact mechanism is unclear, but a cell population with memory may be involved to evoke a similar dermatitis in an exact distribution, such as TRMs. Similarly, taxanes are known to induce photo or UVR recall reactions, where reactivation of solar erythema or solar radiation burns occurs after taxane exposure.^2^ TRMs have also been implicated in this process^6^ and UVR is known to modulate and influence the population of TRMs.^7^ Finally, given that UVR can be involved in the pathogenesis of photodistributed cutaneous DM and that our patient’s docetaxel-associated recurrent DM lesions were photodistributed, we hypothesize that akin to UVR recall, photodistributed lesional TRMs were reactivated by docetaxel, leading to clinical recurrence of cutaneous DM. This is further supported by his lack of both recurrent clinical myositis and spontaneous resolution between infusions.

We favor that this is a recall effect, but that diagnosis could also be considered a drug reaction given that the recall was docetaxel related. We would also ask whether prior reports of taxane-induced DM were truly drug-induced or if they helped to unmask or recall subclinical DM. Regardless, since his last docetaxel infusion, the patient has been in remission from both his DM and malignancy. Additionally, this case highlights the recommendation to perform whole-body imaging in adult DM patients with anti-TIF-1γ positivity due to malignancy risk, as opposed to only age-appropriate cancer screenings.^1^ It is worth noting that metastatic prostate cancer presenting with a normal or negative prostate-specific antigen is rare and patients tend to have poorer outcomes.^8^ In this patient with metastatic prostate cancer and a normal prostate-specific antigen, had whole body imaging not been performed, his malignancy may not have been detected, delaying treatment.

## Conflicts of interest

None disclosed.
